# Studies on the antifungal and serotonin receptor agonist activities of the secondary metabolites from piezotolerant deep-sea fungus *Ascotricha* sp.

**DOI:** 10.1080/21501203.2018.1541934

**Published:** 2018-11-21

**Authors:** A. Ganesh Kumar, K. Balamurugan, R. Vijaya Raghavan, G. Dharani, R. Kirubagaran

**Affiliations:** a Marine Biotechnology Division, Ocean Science and Technology for Islands Group, ESSO – National Institute of Ocean Technology, Ministry of Earth Sciences (MoES), Government of India, Chennai, Tamilnadu, India; b Department of Biotechnology, Alagappa University, Karaikudi, Tamilnadu, India

**Keywords:** *Ascotricha* sp, *Caenorhabditis elegans*, *Candida albicans*, sesquiterpenes, 5-HT_2C_ receptor agonist

## Abstract

The potent antifungal agent sesquiterpenes and serotonin 5-HT_2C_ agonist ascotricin were produced by a newly isolated deep-sea fungus *Ascotricha* sp. This fungus was isolated from deep-sea sediment collected at a depth of 1235 m and characterized. Piezotolerance was successfully tested under high pressure-low temperature (100 bar pressure and 20ºC) microbial cultivation system. Production of secondary metabolites was enhanced at optimized culture conditions. The *in-vivo* antifungal activity of sesquiterpenes was studied using the *Caenorhabditis elegans – Candida albicans* model system. The sesquiterpenes affected the virulence of *C. albicans* and prolonged the life of the host *C. elegans*. These findings suggest that sesquiterpenes are attractive antifungal drug candidates. The 5-HT_2C_ receptor agonist is a potential target for the development of drugs for a range of central nervous system disorders. The interaction of 5-HT_2C_ agonist ascotricin with the receptor was studied through bioinformatic analysis. The *in silico* molecular docking and molecular dynamic simulation studies demonstrated that they fit into the serotonin 5-HT_2C_ active site and the crucial amino acid residues involved in the interactions were identified. To our knowledge, this is first report of *in vivo* antifungal analysis of sesquiterpenes and *in silico* studies of serotonin 5-HT_2C_ receptor-ascotricin complex.

## Graphical Abstract

**Figure:**
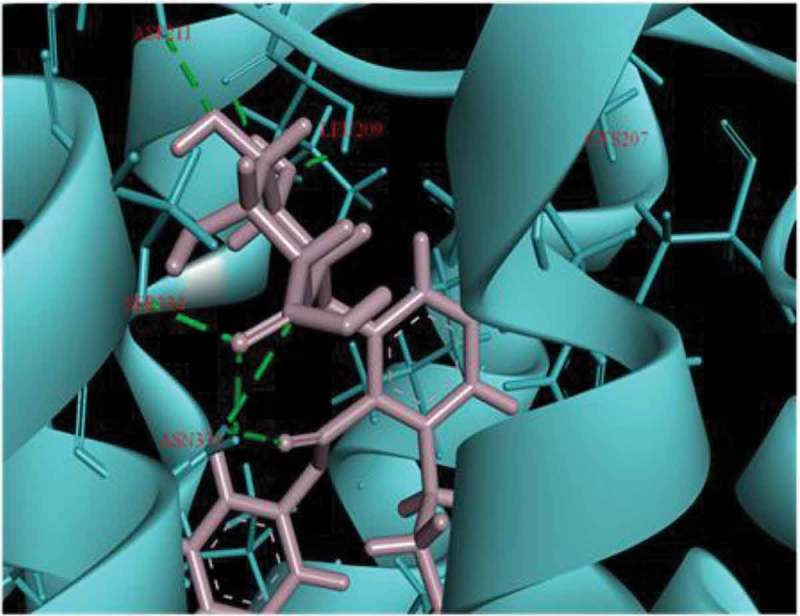

MD simulation of interaction of ascotricin with 5-HT2C receptor

## 1. Introduction

Secondary metabolites of fungal origin have numerous industrial and pharmaceutical applications. Till date, approximately 22,500 biologically active compounds have been obtained from fungi. New compounds are required to combat the development of resistance in microbes and to treat life-threatening diseases such as cancer (Demain and Sanchez 2009). Although considerable progress is being made, the deep-sea still remains the richest source of new bioactive compounds which are yet to be explored. In particular, fungi are eukaryotes having the ability to develop specialized structures allowing them to inhabit and survive all ecological niches. Fungi are the significant source for new and/or bioactive metabolites depending on the culturing growth conditions. Some of these metabolites have adverse toxic activities, e.g. aflatoxin produced by *Aspergillus flavus* (Klich 2007) or useful natural products as drugs (Li and Vederas 2009). Fungi have the ability to produce diverse secondary metabolites typically at a particular period in the life cycle that coincides with the development of the fungus (Calvo et al. 2002). Fungi belonging to the genus *Ascotricha* are capable of producing a diverse array of secondary metabolites. A lanostane-type triterpenoid ascosteroside with antifungal property was isolated from *Ascotricha amphitricha* ATCC 74,237 (Leet et al. 1996). A cyclonerodiol analogue 3,4-seco lanostane triterpenoid was produced by a marine-derived fungus *Ascotricha* sp. ZJ-M-5 (Wang et al. 2014a). An antagonist of sphingosine-1-phosphate receptor 1 was isolated from *Ascotricha chartarum berk* SANK 14,186 (Yonesu et al. 2009). Development of novel antifungal drug is vital, since, in the recent years, considerable case of pathogenic fungi with different drug-resistant patterns have been observed. This is interesting because antifungal resistance emerging from environmental sources was observed in *Aspergillus fumigates* (Sanglard 2016). Moreover, the acquired resistance to antifungal drugs and development of multidrug resistance becomes a major problem in *Candida* species (Sanguinetti et al. 2015). Multiple factors which enable pathogenic fungi to attach, colonize and establish within the host system are yet to be explored. Most of the pathogenic fungi are dimorphic and have the ability to switch their morphology between both the yeast and filamentous forms. This inter-switching mechanism alters the host-microorganisms interactions and it plays a captious role to exert pathogenicity. The fungus *Mucor rouxii* elicits two different morphogenetic phenomena: mycelium or yeast cells based on growth factors and environmental conditions (Lucio et al. 2000). The *Candida albicans* exhibits a versatile morphogenesis and exist in yeasts, pseudohyphae and hyphae forms. The major transition phase from yeast to hyphal form contributes for its pathogenicity facilitating the invasion of host tissues (Sudbery 2011). The free-living nematode *Caenorhabditis elegans* based model system for antifungal drug discovery allows the study of dynamics of host-pathogen interactions *in vivo*. This analysis also provides insights into the novel drug targets, to inhibit fungal pathogen and underlying molecular mechanisms (Anastassopoulou et al. 2011). The serotonin (5-HT) receptor, precisely the 5-HT_2C_ receptor is an emerging and promising drug target to treat several disorders of the human central nervous system-CNS (Neelamegam et al. 2014). The process of development and targeting the 5-HT_2C_ system is of great challenge due to lack of selectivity over 5-HT_2A_ and 5-HT_2B_ receptors. The FDA approved 5-HT_2C_ agonist lorcaserin also triggers 5-HT_2A_ and 5-HT_2B_ receptors activity (Clinton et al. 2014). These results support further the need for development of specific 5-HT_2C_ agonist.

In recent years, deep-sea fungi have been extensively explored for the discovery of new compounds with novel biological properties. Although there are reports on marine fungus and secondary metabolites production, there are only few known applications of piezophiles or piezotolerant deep-sea fungus (Ganesh Kumar et al. 2015). This is due to the fact that it is not easy to cultivate piezophiles under high-pressure conditions using current technology. Bioactivity-guided fractionation of *Ascotricha* sp. extracts led to the isolation of two compounds, including sesquiterpenes and ascotricin. Sesquiterpenes was evaluated for antifungal activity and ascotricin was evaluated for serotonin receptor agonist activity. At the best of our knowledge, this is possibly the first report in which piezotolerant *Ascotricha* sp. has been isolated from deep-sea sediments and the potential of secondary metabolites activity both under *in vitro, in vivo* and *in silico* conditions have been unveiled for the discovery of new antifungal agent and 5-HT_2C_ receptor-specific agonist.

## 2. Materials and method

### 2.1. Sample materials and fungus isolation

Deep-sea fungus was isolated from sediment samples collected from deep-sea at a depth of 1235 m from Off-Cochin (10° 21´117´ N 75° 47´ 252´ E). The growth and purification of the isolate were performed on potato dextrose agar (PDA), which was comprised of 200 g potato, 20 g dextrose and 15 g agar in 1000 mL filter sterilized seawater. The culture was stocked at 4°C and subcultured once in every 3 weeks.

### 2.2. Genomic DNA isolation and PCR amplification

For genomic DNA extraction the fungus was cultured on the PDB at 28ºC for 5 days. The mycelia mat was transferred to 1.5 mL tube and DNA was extracted using high salt method. The 18S rRNA was amplified using Euk 18S 555F – Euk 18S 1269R primers and the ITS region was amplified using ITS1 – ITS2 primers. Total reaction volume 20 µL composed of 10 µL PCR master mix (Takara), 1 µL forward and reverse primers (10 pmol), 2 µL genomic DNA (3 ng), 1 µL of filter-sterilized DMSO and 5 µL of nuclease-free water. PCR products were purified and sequenced using an automated sequencer.

### 2.3. Nucleotide sequences and phylogenetic analysis

The DNA sequences were compared to the reference sequences within the NCBI database using the BLASTN algorithm. All the chosen relative sequences were aligned using BIOEDIT ver.7.2.5 (Hall 1999). Then the data set were analysed using maximum likelihood in MEGA 7.0 to determine the best-studied nucleotide substitution model. Phylogenetic tree along with an outgroup was generated using MEGA 7.0.

### 2.4. Characterization of piezotolerance

To examine the piezotolerance, the isolate was incubated at 100 bar pressure at 20ºC using customized pressure vessels (ESSO-NIOT, India) simulating *in situ* deep-sea conditions. In stationary growth phase, the cells were harvested by centrifugation at 8000g for 15 min. The isolate was cultivated on PDB (½ concentration) prepared in sea water and incubated at 10 bar pressure in 20ºC for 1–2 days, after which pressure was increased gradually up to 100 bar for 7–10 days. The growth of the fungus was examined in phase contrast microscope (Carl Zeiss Axioskop 2 plus, Carl Zeiss Microscopy LLC, USA) after exposure to elevated pressure conditions.

### 2.5. Optimization of culture growth and secondary metabolite production

Optimization of parameters such as temperature (4, 10, 20, 30 and 40ºC), pH (5.0, 6.0, 7.0, 8.0, 9.0 and 10.0) and salinity (2%, 4%, 6%, 8%, and 10%) were tested in potato dextrose broth (PDB). The effect of carbon source (dextrose, sucrose, maltose, starch and cellulose) at a concentration of 1% and the effect of nitrogen source (ammonium sulphate, ammonium dihydrogen phosphate, ammonium nitrate, yeast extract, beef extract, peptone, tryptophan, tyrosine and phenylalanine) at a concentration of 20 mmol L^−1^ in minimal salt medium (MSM) with the following composition: 1gL^−1^ NaCl, 0.1 g L^−1^ KCl, 1gL^−1^ KH_2_PO_4_, 1.5gL^−1^ K_2_HPO_4_, 0.3 g L^− 1^ MgSO_4_ · 7H_2_O (per liter of 1:1 seawater and distilled water). To this, filter sterilized trace element solution (20 mgL^−1^ CaCl_2_, 30 mgL^−1^ FeCl_3_, 0.5 mg L^−1^ CuSO_4_.5H_2_O, 0.5 mg L^−1^ MnSO_4_.H_2_O, 10 mg L^−1^ ZnSO_4_.7H_2_O) and carbon source 20 g L^− 1^ dextrose were added to the pre-sterilized MSM media to evaluate the nitrogen source influencing bioactive compounds production. The flasks were inoculated with 10% of 5-day old fungal broth culture. The flasks were then incubated in dark under shaking conditions (120 rpm) at 28 ± 2ºC for 7 days. The fungal biomass growth was monitored by measuring mycelia dry weight and extracellular protein. Metabolic activities were monitored by measuring the reducing sugars and secondary metabolites every day up to 7 days and all analyses were carried out in duplicates.

### 2.6. Fermentation and extraction

Pure culture slant was inoculated into the PDB (Difco, Becton Dickinson and Company, France) prepared in sea water and incubated at 28ºC under shaking conditions for 7 days. The secondary metabolites were extracted from the culture broth with an equal volume of n-butanol. The organic layer was separated and concentrated in a rotary evaporator (R215, BUCHI-Switzerland). The n-butanol extract obtained from culture broth was subjected to a Silica Gel GC 60–120 mesh column chromatography using chloroform/methanol 90:10–50:50 solvent systems to yield different fractions. All the fractions were tested against two type strains, namely, *Amylomyces rouxii* MTCC 386 and *Aspergillus fumigatus* MTCC 2550 (Microbial Type Culture Collection, IMTECH, Chandigarh).

### 2.7. Purification and structure elucidation

A milligram of bioactive subfraction was dissolved in MeOH and filtered through 0.22 µm syringe membrane filter. This filtrate was then subjected to HPLC analysis on an Agilent C18 column (5 µm, 150 mm × 4.6 mm i.dd.) at a flow rate of 1.0 mL/min. The elution gradient (methanol-water) was setup as 0 ~ 5 min, 5% as 0 ~ 5 min, 5%; 5 ~ 65 min, 5%~100%; 65 ~ 75 min, 100%B; 75 ~ 80 min, 100%~5%B. Bioactivity-guided fractionation of extracts led to the isolation of two compounds, colourless powder (compound I) and solvent fraction (compound II). ^1^H spectra and ^13^C-NMR spectra for compound I (colorless powder) in DMSO-d6 was recorded at 40ºC in high-resolution liquid state NMR (Jeol ECA 500 MHz). ESI-MS analysis was performed using Agilent 6545 Q-TOF mass spectrometer. The FTIR analysis was carried out in the range of 4000 to 400 cm^−1^ with Schimadhzu IR affinity spectrometer. The solvent fraction (compound II) was dissolved in 0.5 mL methanol and injected into the GC-MS instrument (Agilent Technologies GCMS 5973, USA), and the separated compounds were identified using library match.

### 2.8. Determination of the minimum inhibitory concentration (MIC)

The solvent fraction (compound II) was evaluated for antifungal activity against test pathogens. The test fungi used in the present study including *Cryptococcus albidus* (MTCC 4746), *Candida albicans* (MTCC 227), *Aspergillus fumigatus* (MTCC 2550), *Aspergillus flavus* (MTCC 277), *Penicillium chrysogenum* (MTCC 2725), *Amylomyces rouxii* (MTCC 386), *Aspergillus terreus* (MTCC 3572), *Aspergillus niger* (MTCC 282), *Trichophyton rubrum* (MTCC 296) and *Saccharomyces cerevisiae* (MTCC 307) were from the Institute of Microbial Technology (IMTECH), India. The test compound was dissolved in DMSO (5%) at a final concentration of 10 mg/mL, and serial dilutions were prepared using the same solvent. The MIC was determined on 96 well microdilution plates according to the standard protocols ((NCCLS) National Committee for Clinical Laboratory Standards 1997) incubating at 28ºC for 48 h. Microorganisms were exposed to serial dilutions of the compounds keeping a final concentration <5% in each well to avoid DMSO toxicity. Endpoints were determined when no turbidity in the well was observed. The antifungal activities of the compounds were compared to amphotericin B. All assays were carried out in triplicate.

### 2.9. In vivo C. elegans toxicity analysis

Age-synchronized young-adult stage *C. elegans* were grown in nematode growth medium (NGM) in petri dishes and fed with *E. coli* OP50 as food source. Three different (sesquiterpenes) metabolites concentration were tested 100 ng, 10 µg and 100 µg using a balanced salt solution (M-9) of composition 1.5 g KH_2_PO_4_, 3.0 Na_2_HPO_4_, 2.5 g NaCl, 0.5 mL 1M MgSO_4_ in 500 mL distilled water (Brenner 1974). A test was performed in 12 well plates containing a total volume of 1 mL/well with triplicates per treatment. The young-adult worms 10 numbers were introduced per well and incubated at 25ºC. The life/health-span of the animals was monitored for their growth and developments for every 24 h under a microscope (Durai et al. 2013). The antifungal drug amphotericin B was used as positive control.

### 2.10. In vivo antifungal analysis

A previously described protocol for infecting *C. elegans* with *C. albicans* in a liquid medium pathogenesis assay was modified for these studies (Breger et al. 2007). Freshly grown *C. albicans* cells were inoculated into 1 mL of yeast extract-peptone-dextrose and allowed to grow overnight at 30ºC. The following day, 100 µL of yeast was spread into a square lawn on a 10 cm plate containing BHI agar and kanamycin (45 µg/mL), following by incubation for approximately 20 h at 30ºC. Kanamycin was added to prevent growth of any residual *E. coli* OP50 strain carried on the nematode during the experiment. For control experiments using only *E. coli* OP50, kanamycin was omitted from the liquid medium. Worms were scored daily into one of three categories: alive, dead with hyphae piercing the cuticle, and dead without hyphae piercing the cuticle. Worms were considered dead if they did not move in response to mechanical stimulation with a pick. Dead worms were removed from the assay. Microscopies of nematodes were performed by using Zeiss Axio Imager microscope.

### 2.11. Molecular docking and molecular dynamics simulation of ascotricin A

The compound I (ascotricin A) was evaluated for *in vitro* agonist activity as described earlier with minor modification (Lambert and Lauder 1999). The docking of ascotricin A with 5-HT_2C_ receptor was carried out using the CDOCKER module for Discovery studio V4.0 (DS). The structure of the receptor 5-HT_2C_ was obtained from protein data bank (PDB Id: 6BQG). The active site was predicted and docking was carried out subsequently. Best poses with lowest energy values associated with favourable region of binding were obtained. Molecular dynamics (MD) simulation studies were carried out using the MD simulation module in DS. The atoms were typed according to CHARMM force field and were solvated. The surface charges were neutralized by adding Na^2+^ and Cl^2-^ counterions. Prior to simulation, the system was energy minimized using steepest descent and conjugate gradient methods. The system was equilibrated with a time step of 2 fs at a temperature of 300 K and 1 atm pressure. The production run was carried out for a time period of 100ps and the trajectories were saved for every 1ps. The RMSF, total potential energy of the system and other parameters were analysed using the various modules of DS. The crucial residues that were involved in the interaction between ascotricin A and 5-HT_2C_ receptor were further studied based on the conformations generations through MD simulation.

## 3. Results

### 3.1. Morphological and molecular characterization of deep-sea fungus

**Figure 1.**
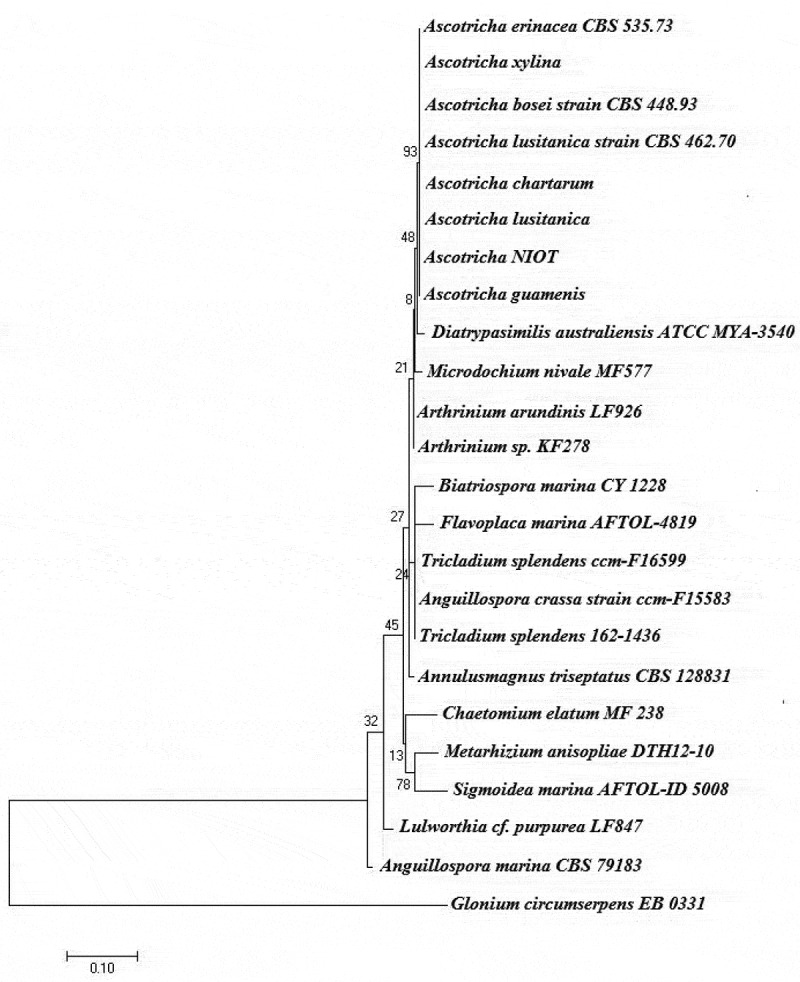
Phylogenetic placement of deep-sea derived *Ascotricha* sp. using the maximum-likelihood method based on the Tamura-Nei model. Bootstrap values were indicated at nodes based on a neighbour-joining analysis of 1000 replicates. Scale bar was equal to 0.1 substitutions per nucleotide position.

The fungus producing bioactive compound was identified as *Ascotricha* sp. based on the combination of morphological characteristics including ascomata, ascomatal hairs, conidiophores and spores. The mycelium of the isolate produced yellow tint mat-like colonies on potato dextrose agar at 28ºC and changed to dark green on sporulation. The monoculture matured within 144 h with sporulation phases and produced secondary metabolites extracellularly. The molecular characteristics including 18S rRNA and ribosomal intergenic spacer region (ITS) confirmed that the deep-sea fungus belongs to the genus *Ascotricha* sp. and is most closely related to *Ascotricha lusitanica* showing 98% homology similarity. The sequences were imported to BIOEDIT ver.7.2.5 (Hall 1999) and aligned using ClustalW. The evolutionary history was depicted as a phylogenetic tree employing Maximum likelihood (ML) method based on the Tamura-Nei (TN) nucleotide substitutions model as selected by MEGA 7.0. Phylogenetic tree was generated using MEGA 7.0 with bootstrap values calculated from 1000 replicates (Figure 1). The 18S rRNA and ITS sequences were deposited in NCBI, under accession number KY654745.1 and KY654751.1.

### 3.2. Piezotolerance analysis

**Figure 2.**
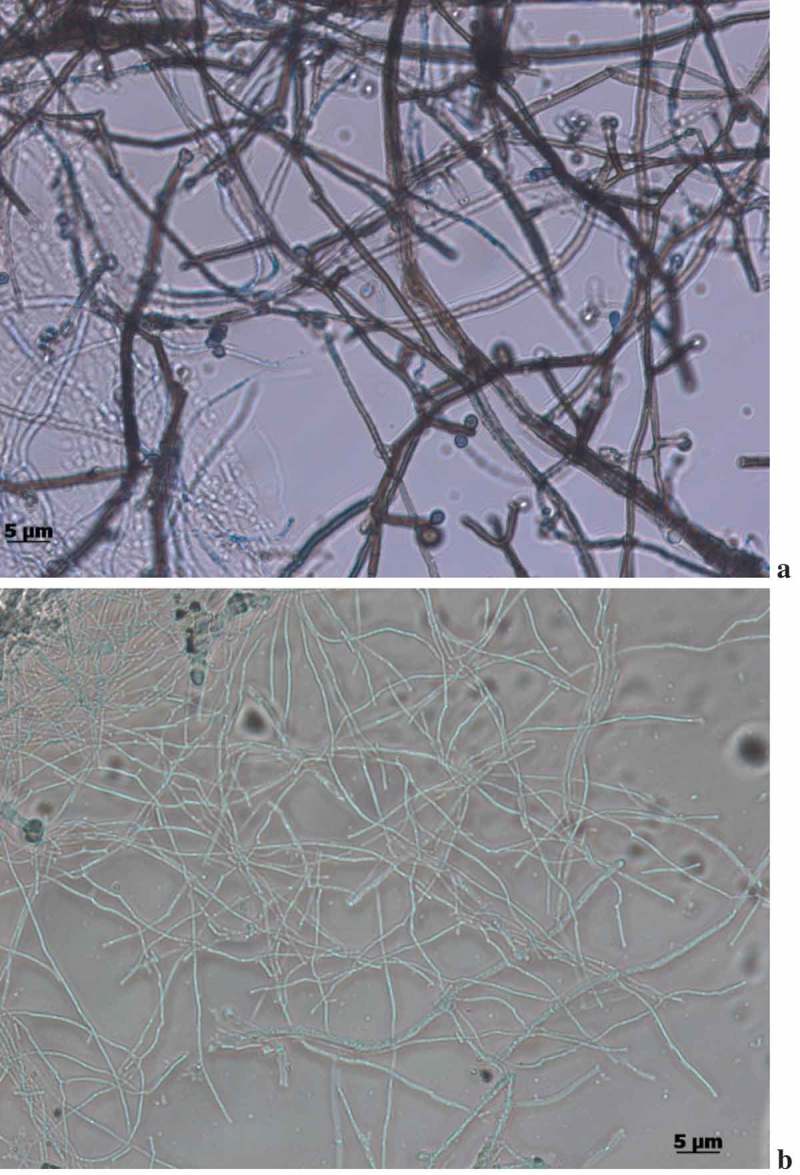
Morphological conditions of *Ascotricha* sp. grown at a. 1 bar. b. 100 bar pressure.

This is the first study to report on the isolation of *Ascoticha* sp. from deep-sea sediment collected at 1235 m from Off-Cochin (India). Microscopical analysis elucidated the pressure induced alterations in the morphology. Much reduced mycelial hyphae were observed in fungus grown at 100 bar pressure. The size was reduced to 4–6 times in cells grown at elevated pressure compared to the growth at 1 bar pressure (Figure 2(a-b)). Although the growth rate was reduced the growth was not inhibited by the increase in pressure. Interestingly, the fruiting ascomata were not observed at 100 bar pressure. The ability of *Ascotricha* sp. to grow at elevated pressure conditions elucidated its piezotolerance potential.

### 3.3. Growth of Ascotricha sp.

The growth *Ascotricha* sp. showed a log phase from 48-120h and this phase were followed by a prominent decline in the mycelia biomass until 168 h. In submerged culture this fungus exerted accelerated growth metabolism and showed high mycelial production. The amount of secondary metabolites remained significantly higher than in the log phase. Therefore, it was a capable fungus with fascinating features.

### 3.4. Effect of pH, temperature and salinity on growth and metabolites synthesis

*Ascotricha* sp. was cultured in the flask at different pH (5.0–10.0) conditions. Our results elucidated the potential of strain to grow and produce the secondary metabolites at a broad spectra of pH ranging from 5.0 to 9.0. This fungus showed the highest growth of culture biomass (31.3 g/L) and production of secondary metabolites (1.14 g/L) when incubated in PDB for 120 h with an initial pH value of the medium at 9.0. Thus, the pH 9.0 was maintained in fermentation medium throughout the experimental studies. The effect of temperatures at 4, 10, 20, 30 and 40ºC on growth and metabolites synthesis by *Ascotricha* sp. was studied with pH 9.0. The growth of fungus increased while the temperature was increased from 10 to 30ºC, both biomass (30.9 g/L) and secondary metabolite production (1.12 g/L) was estimated to be highest at 30ºC. High growth and metabolites synthesis were observed at both 2% and 4% salinity concentration. The mycelial biomass (36.8 g/L) and secondary metabolites (1.29 g/L) production were observed to reach a peak value at 4% salinity followed by biomass (34.1 g/L) and secondary metabolites (1.01 g/L) production at 2% salinity concentration. The mycelia of *Ascotricha* sp. grew well at 0% salinity however it affected the biosynthesis of secondary metabolites.

### 3.5. Effect of carbon and nitrogen sources

The mycelia dry weight, pH values, total protein and extracellular secondary metabolites of the culture filtrates of *Ascotricha* sp. was examined in the basal medium with various carbon sources, namely, dextrose, sucrose, maltose, starch and cellulose. Among the tested sources, sucrose gave significantly highest mycelium biomass (33.15 g/L) and bioactive metabolites production (0.92 g/L). Second high mycelium (26.6 g/L) was observed in dextrose but the secondary metabolites (0.66 g/L) were produced at moderate level. This was followed by maltose, starch and cellulose, which were considered as a poor source of carbon for the growth of this isolate. The cellulose was the least utilized carbon source and retarded growth was observed. Thus sucrose was provided as the sole carbon source for further studies. Nitrogen is an important factor influencing not only fungal growth and differentiation, but also on the secondary metabolites biosynthesis. Effects of different nitrogen sources (inorganic and organic) such as ammonium sulphate, ammonium dihydrogen phosphate, ammonium nitrate, yeast extract, beef extract, peptone, tryptophan, tyrosine and phenyl alanine on fungus growth and secondary metabolites production were analysed. Various nitrogen sources stimulated the growth of the *Ascotricha* sp. and some sources increased the production of secondary metabolites. Based on the inorganic nitrogen sources tested, the ammonium sulphate was the best nitrogen source and it supplementation significantly increased the mycelia biomass (34.4 gL^−1^) and secondary metabolites (0.86 gL^−1^) production. In addition, among the organic nitrogen sources tested, peptone influenced the biomass (31.5 gL^−1^) and secondary metabolites (0.92 gL^−1^) production. Interestingly, the addition of aromatic amino acid phenylalanine stimulated both the mycelial biomass (34.2 gL^−1^) and secondary metabolites (1.17 gL^−1^) biosynthesis on day 5 in the culture medium.

### 3.6. Purification and structure elucidation

**Figure 3.**
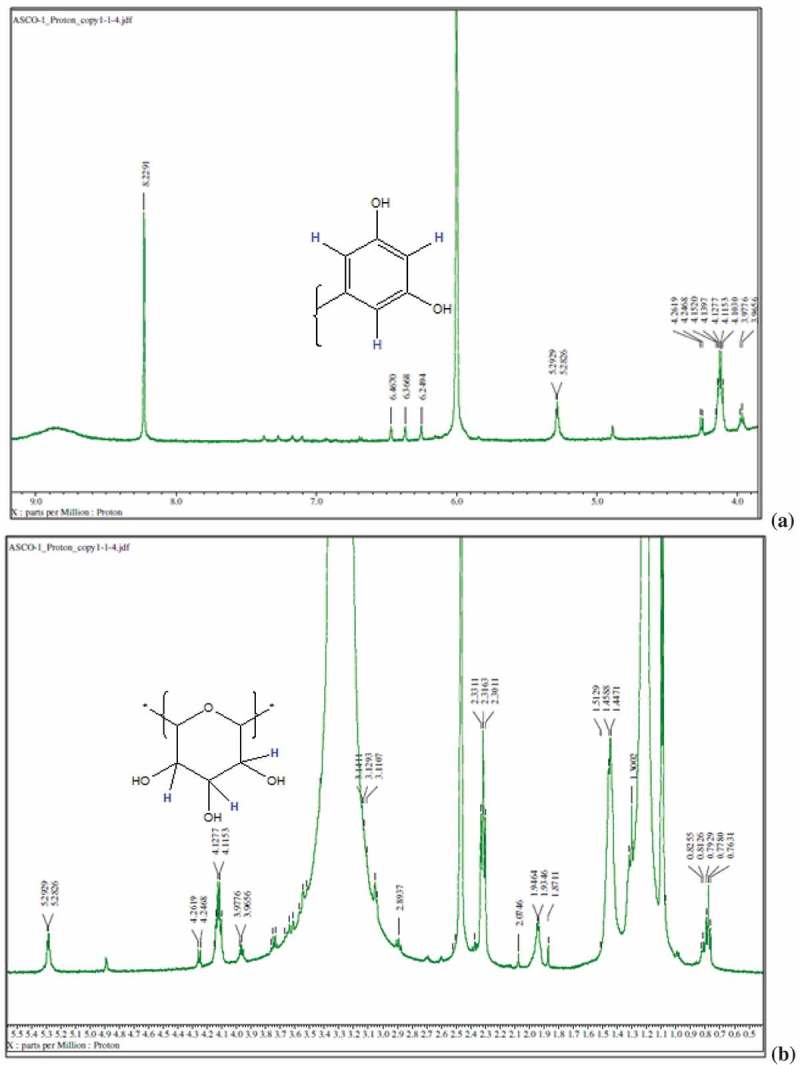
^1^H NMR spectra of compound I (ascotricin A).

**Figure 4.**
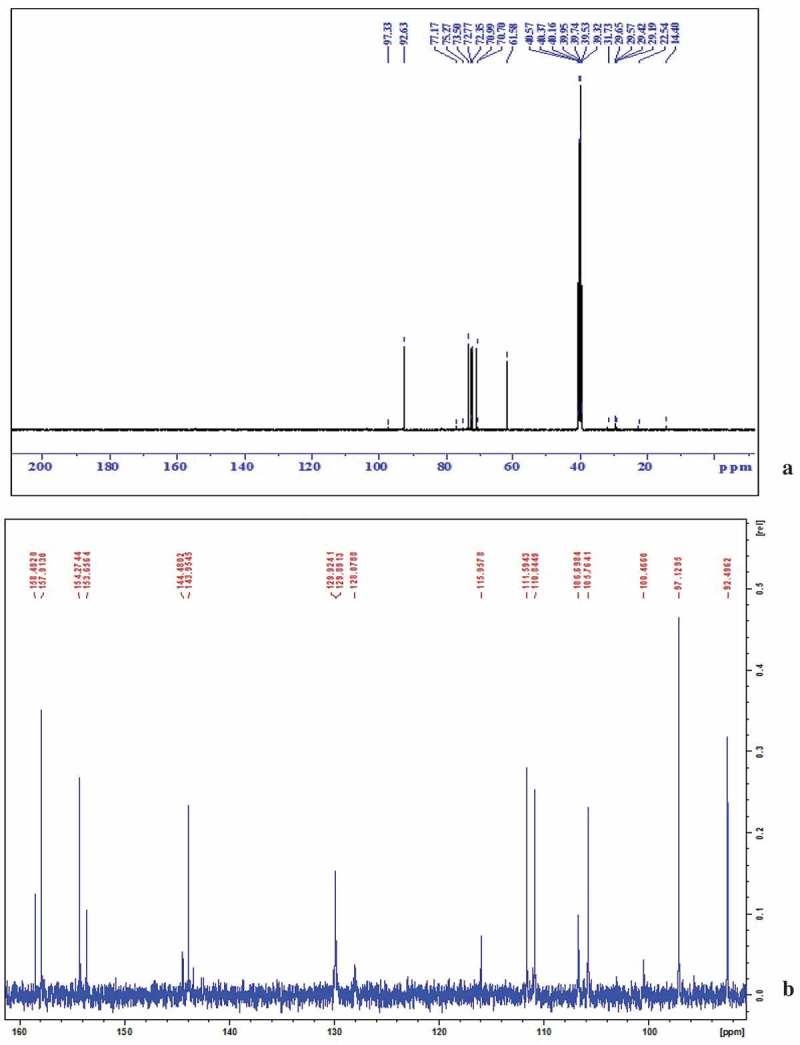
^13^C NMR spectra of compound I (ascotricin A).

**Figure 5.**
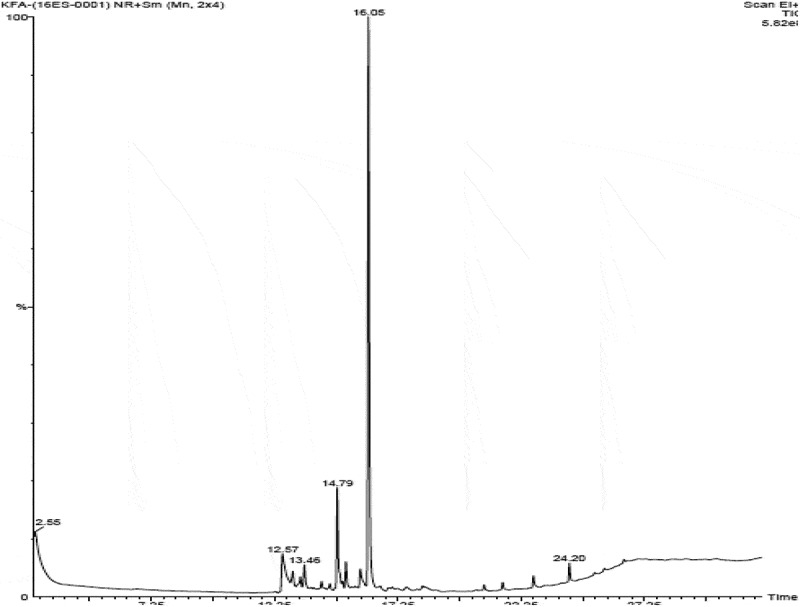
GC-MS analysis of the extracellular secondary metabolites.

Bioactive subfractions yielded compound I (colourless powder) and compound II. The HPLC analysis of compound I confirmed the presence of single dominant peak and structural elucidation was carried by FTIR, ^1^H NMR, ^13^C NMR, and mass spectroscopic analysis. The FTIR spectra showed the presence of carboxylic acid group at 1734 cm^−1^ and alkyl groups at 2950 and 2922 cm^−1^ (Supplementary File 1). The ^1^H NMR spectra of compound I (colourless powder) elucidated the presence of aromatic protons and hydroxyl peaks (Figure 3(a,b)). The ^13^C NMR spectrum confirmed the presence of methylene and aromatic peaks (Figure 4(a,b)). These groups and peak positions were similar to ascotricin-A molecule as reported earlier (Yonesu et al. 2009). The ESI-MS *m/z* of compound I was found to be 557.19 [M +Na]^+^ (Supplementary File 2). The GC-MS analysis of the bioactive compound II yielded more than 4 molecules such as diphenylmethane, 1,6-Cyclodecadiene, sesquiterpenes and dihydro-cis-alpha-copaene-8-ol. The major compound was identified as sesquiterpenes which constituted about 66% (Figure 5).

### 3.7. In vitro and in vivo antifungal analysis of sesquiterpenes

**Figure 6.**
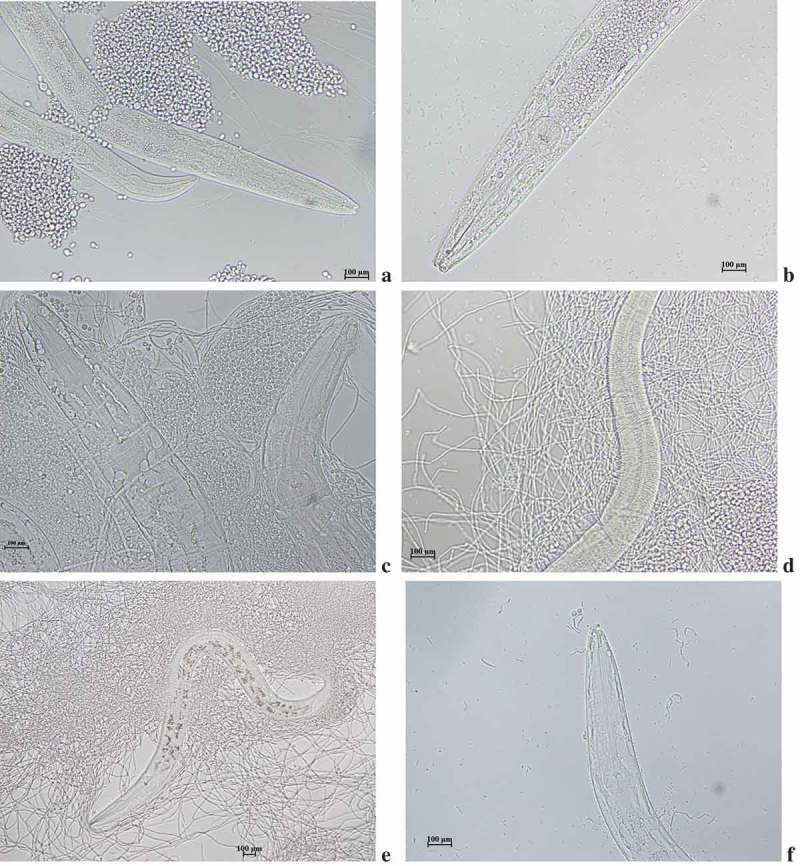
Photomicrograph of (a) *C. albicans* pre-inoculted with *C. elegans*. (b) accumulation of yeast cells inside nematode (c) Transformation of yeast to mycelia forms (d) Cuticle damage in nematode (e) Arrest of nematode movement (f) Incomplete transformation of yeast to mycelial phase.

The sesquiterpenes (compound II) inhibited the growth of fungi and MIC was found to be 4.0 µg/mL – *Mucor rouxii*, 6.0 µg/mL *– Aspergillus fumigates* and 5.0 µg/mL *– Candida albicans*. No activity was found against *Aspergillus spinulosus, Aspergillus terreus* and *Peniclillium chrysogenum*. Using *C. elegans-C. albicans* model system (Figure 6(a)), the *in vivo* antifungal activity of HPLC fractionated bioactive sesquiterpenes was tested. The yeast phase *C. albicans* accumulateed inside the nematode by following adaptive strategies to evade host defence (Figure 6(b)). This is followed by transformation of vegetative yeast phase into pathogenic mycelia forms (Figure 6(c)). The phenotypic plasticity of *C. albicans* rendered remarkable morphological transformation and produced elongated hyphae damaging the cuticles (Figure 6(d)). These transformations also arrested the activity of uninfected nematodes providing an opportunity for *C. albicans* infection (Figure 6(e)). Bioactive fractions provided more than 70% survival rate in fungal infected nematodes. Confirming the *in vitro* analysis the sesquiterpenes promoted the fungus infected nematodes survival. Interestingly, the dose response for nematode survival for sesquiterpene compound was comparable to amphotericin B. At high concentration, the beneficial effect of secondary metabolites was lost and the nematodes died faster than untreated worms. Our compound reverted pathogenic filamentous *C. albicans* forms to vegetative yeast forms in presence of secondary metabolites at concentration of 10 µg/mL (Figure 6(f)). The sesquiterpenes up to a concentration of 20 µg/mL was effective in prolonging the survival of nematodes exposed to *C. albicans* MTCC, but at higher concentration (50 µg/mL) nematode survival was diminished, even compared to the nematodes in the untreated control group. Probable this toxicity is present at even low concentrations, but the beneficial effect from the antifungal outweighs the toxic effect.

### 3.8. Evaluation of compound toxicity in C. elegans

This screening allowed assessment of the compound toxicity level based on *C. elegans* survival at MIC compound concentration. In the initial phase of the curve within 12 h, there was death of nematodes in both positive control and test sample. Time-dependent toxicity was not observed as the nematodes entered a strong recovery phase and stabilized with time intervals. From this result, it was confirmed that secondary metabolites exerted short-term toxicity causing death of the *C. elegans* with minimal concentration (10 µg/mL) followed by stabilized phase of recovery.

### 3.9. Molecular docking of ascotricin

**Figure 7.**
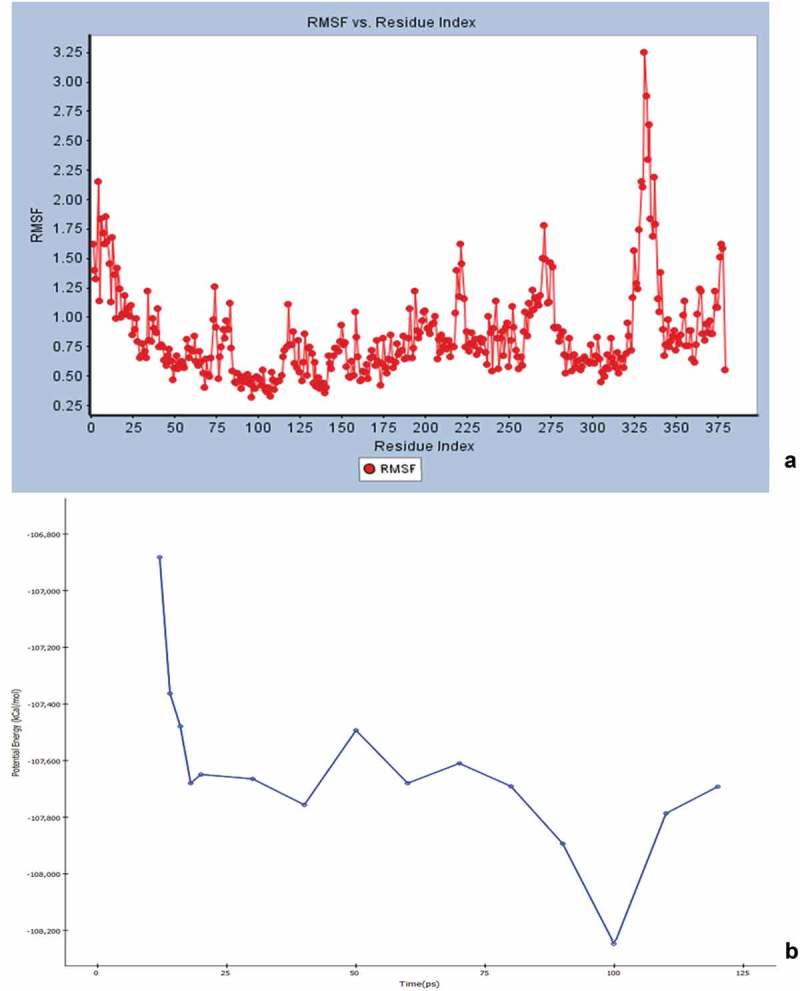
(a) The root mean square fluction (RMSF) of the 5-HT_2C_ residues over the simulation time period is indicated. (b) The change in total potential energy of the receptor ligand complex over the due course of MD simulation is indicated.

Initial screening confirmed the human serotonin 5-HT_2C_ agonist activity of compound I (ascotricin) and further attempts were made to elucidate the molecular interactions between the molecule and the receptor. Molecular Docking studies confirmed ascotricin A and 5-HT_2C_ docked with a binding energy of −47.34 kcal/mol. The results indicated that the residues ASN331, SER219 and VAL215 formed favourable hydrogen bond with ascotricin A and the residues LEU209, TRP324, PHE327, VAL354 were involved in hydrophobic interaction. Conformations generated using MD simulations suggested that the binding energy of ascotricin A and 5-HT_2C_ complex has increased further to −62.5827 kcal/mol indicating more compact binding in the active site. The plots indicating the changes in root-mean-square fluctuation (RMSF) and the total potential energy of the system are given in Figure 7.

**Figure 8.**
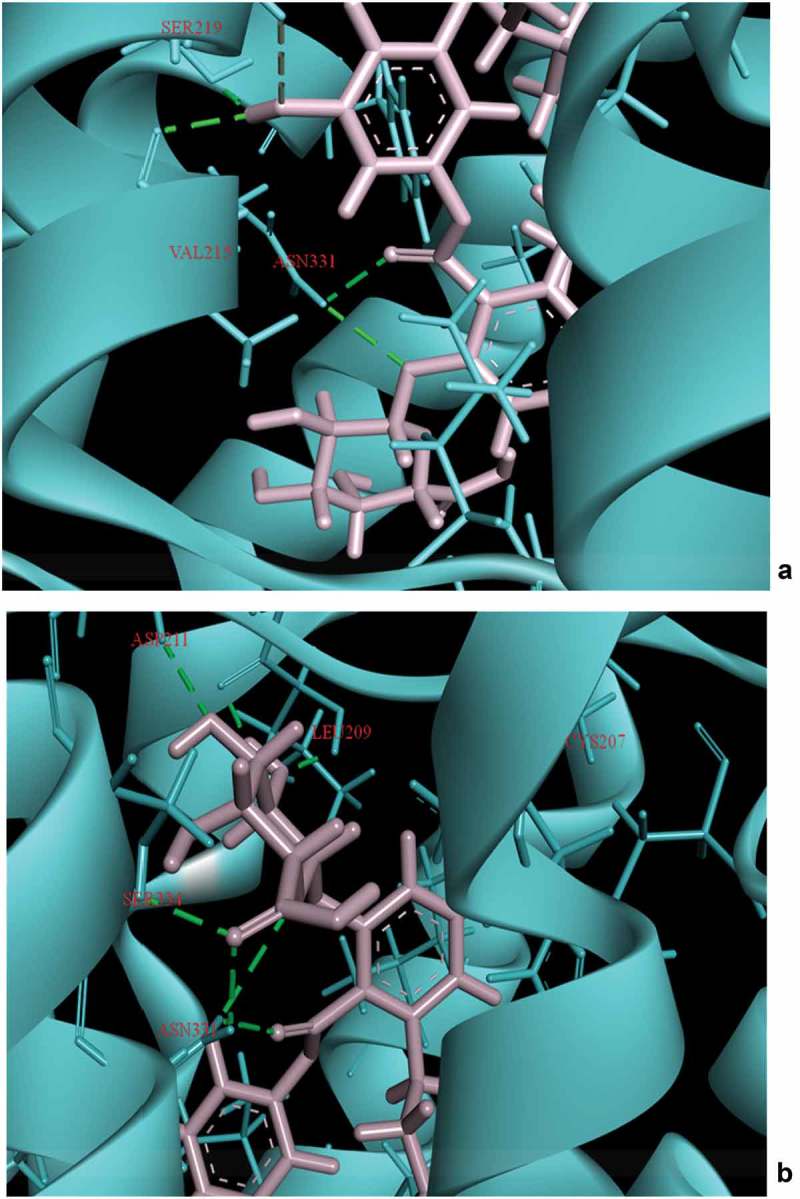
Interaction of Ascotricin A with 5HT2C after (a) molecular docking. (b) 100ps MD simulation. The residues that form hydrogen bond interactions with ascotricin A are indicated (green dotted lines).

Post-MD simulations, the residues LEU209, ASP211, SER334, CYS207, SER219 and ASN331were found to form hydrogen bonds with ascotricin A (Figure 8). The number of residues involved in hydrogen bonding had increased post-MD simulation, a major factor in more stringent binding. The residues ASN331 and SER219 were involved in the interactions under static as well as dynamic conditions. The hydrogen bond between the carboxyl group of the ligand and the NH group of ASN331 and between the oxygen of SER219 and the hydrogen of the ligand were found to be present throughout the simulation time period, presumably the crucial residues in the interaction. The pi-pi stacking was observed only in the residue PHE327 upon docking but after the MD simulations it was observed that the residue TYR118 also formed pi-pi stacking in addition to PHE327. No significant changes were observed in the structure of the receptor after MD simulations. The root-mean-square deviation (RMSD) between the 5-HT_2C_ structure before and after MD simulation was 3.149 Å.

## 4. Discussion

To the best of our knowledge, this is the first report on the *in vitro* and *in vivo* antifungal activity of the sesquiterpene derivatives extracted from the deep-sea piezotolerant ascomycetes fungus *Ascotricha* sp. Marine fungi biosynthesis variety of secondary metabolites with unique structures which have gained much importance for discovering new bioactive molecules, for eg., the antimicrobial agent cephalosporin was extracted and purified from a marine fungus, *Cephalosporium acremonium* (Murti and Agarwal 2010). Deep-sea fungi are still one of the most under-studied marine ecological groups. In this study, deep-sea fungus was isolated from surface sediment collected using gravity corer at a depth of 1235m, off-Cochin. Identification of isolated fungus was achieved using PCR amplification of partial 18S rRNA and ITS amplification. The PCR amplification of partial 18S rRNA and ITS amplification confirmed that the isolate was *Ascotricha* sp., an ascomyctes fungi. Previous studies have been well documented that family Ascomycetes include group of obligate marine fungi that tremendously contribute to deep-sea ecosystem (Bhadury et al. 2011; Wang et al. 2014b). The development of advanced sampling system increased the availability of deep-sea fungi for the screening of novel drugs (Wang et al. 2015). This study was aimed to optimize the use of deep-sea piezotolerant fungi as sources for novel compounds and as cell factories for large scale manufacture of bio-based products. The deep-sea fungi have been classified on the basis of their ability to grow under elevated pressure culture conditions. Piezophilic fungi are those that grow and sporulate exclusively in a pressurised environment, while piezotolerant fungi are those that are able to grow and possibly sporulate in both elevated pressure and atmospheric pressure conditions. The isolate of this study was able to grow in both piezophilic and non-piezophilic conditions, and therefore it was classified as piezotolerant fungi. This deep-sea fungus follows a unique pattern of growth and development under piezophilic conditions. Our experimental results elucidated that the growth patterns of this fungus are divided into (i) acclimatization/adaptation phase (ii) hyphae initiation or lag phase (iii) rapid growth of mycelia or log phase and (iv) decline growth phase. The lag may lengthen to weeks because of the fungus adaptation and molecular switching between the piezophilic and non-piezophilic growth conditions (Abe et al. 1999). This reduced growth rate results in a much reduced slope of the log phase. In this study, *Ascotricha* sp. from deep-sea sediment was isolated and its secondary metabolites were screened. The fungal growth and secondary metabolites production may be influenced by changes in physical-chemical and biological factors, and/or by interactions among these factors. The fermentation conditions such as pH, temperature, incubation time, nutrients availability and phase of potential fungal strain growths (stationary-idiophase and growth-tropophase) significantly affect the secondary metabolism (Elias et al. 2006). The pH of the fungal medium is an vital stimulatory factor for the biosynthesis of secondary metabolites. The secondary metabolites and growth was observed at pH 9.0. Our earlier study also correlated with the production of bioactive secondary metabolites at alkaline pH conditions (Ganesh Kumar et al. 2015). Both biomass (20.9 g/L) and secondary metabolite production (1.1 g/L) was estimated to be highest at 30ºC. The secondary metabolite production (0.1 g/L) was much reduced at 40ºC. High cell growth and enhanced metabolite production in *Aspergillus* strain was observed with increase in incubation temperature from 25 to 30 ºC (Bhattacharyya and Jha 2011). Studies with variable temperature on fungus have shown that low temperatures would slow down the metabolite activity whereas high temperatures would cease the growth (Merlin et al. 2013). The salinity concentration was found to possess profound effect on growth and metabolites biosynthesis in fungi. Our isolate was found to grow at a faster rate at 0% salinity than at higher NaCl concentration; however the yield of secondary metabolites is very less. Similar results have been observed in a marine-derived fungus *Arthinium c.f. saccharicola* (Miao et al. 2006). This study suggests that certain marine fungal isolates might have originated from terrestrial or freshwater sources and under the stimulus of high salinity, the fungus would respond with enhanced production of bioactive metabolites (Miao et al. 2006). These results suggest that our isolate could able to transform between low and high salinity conditions. It is well established that different types of carbon sources exhibits great variations in fungal growth mechanism and the biosynthesis of secondary metabolites (Gao et al. 2007). With minimal salt media as the base, four carbon sources were tested namely dextrose, sucrose, cellulose and starch. Among the tested sources, sucrose was found to be the best carbon source for biomass (26.6 g/L) and bioactive metabolite production (0.46 g/L). The biomass production in dextrose was also high with 55 g/L but the secondary metabolite production was moderate (0.22 g/L). Starch was the least utilized carbon source. Similar findings have been reported with *Aspergillus* sp (Bhattacharyya and Jha 2011). According to the analysis results, the sucrose was selected as the optimized carbon source for the secondary metabolites production and thus used for the subsequent experiments. Both the organic and inorganic nitrogen sources were tested to find its influence on *Ascotricha* sp. growth and secondary metabolites generation. High growth of *Ascotricha* sp. was observed in defined medium with ammonium sulphate as the nitrogen source. This result correlated with the findings of (Tamminen et al. 2015), where specific growth rate of marine fungi *Calcarisporium* sp. was found to be high with ammonium sulphate. Interestingly, the organic nitrogen sources peptone also exhibited higher fermentation efficiency. The addition of aromatic amino acid phenyl alanine induces the synthesis of secondary metabolites. This result concluded that phenyl alanine may provide precursors for the biosynthesis secondary metabolites. The structures were determined by FTIR, ^1^H-NMR, ^13^C-NMR and ESI-MS spectroscopic analysis. Sesquiterpenes are the most unique secondary metabolites of the Ascomyctes family. However, they have been reported from several marine fungi with wide biological peroperties. Marine fungus *Aspergillus ustus* isolated from the marine sponge *Suberites domuncula* produced new drimane sesquiterpenoids (Liu et al. 2009). Endophytic marine fungus *Penicillium expansum* was reported to preoduce four new phenolic bisabolane sesquiterpenoids (Lu et al. 2010). A new sesquiterpene, spartinoxide was isolated from the marine fungus *Phaeosphaeria spartinae* (Elsebai et al. 2010). Three azaphilone sesquiterpenoids were isolated from the marine endophytic fungus *Penicillium chermesinum* (Huang et al. 2011). A new chloro-trinoreremophilane sequiterpene was reported from an Antartic deep-sea derived fungus, *Penicillium* sp. (Wu et al. 2013). A *Penicillium* sp. isolated from a deep-sea sediment sample was reported to produce cytotoxic sesquiterpene quinone (Lin et al. 2012). In our study sesquiterpenes showed good antifungal activity. *Ascotricha* sp. inhibited the growth of *A. fumigatus* and *M. rouxii* on a PDA plate. The fungal growth and antifungal compound production was monitored during 7 days. The antifungal compound production increased every day to reach maximum at 6^th^ day. The culture filtrate collected at 7^th^ day completely inhibited the spore germination and hyphal growth. The *in vitro* activity of sesquiterpenes has been demonstrated against molds, yeasts, and dimorphic fungi using methods as suggested by the Clinical and Laboratory standards institute (NCCLS 1997). The MIC values varied from 2–6 µg/mL depending upon the sensitivity of the test fungi *Mucor rouxii* (4.0 µg/mL), *Aspergillus fumigatus* (6.0 µg/mL) and *Candida albicans* (5.0 µg/mL). The option to treat invasive fungal infections caused by *Aspergillus* and *Mucorales* is limited. However, the morbidity and mortality for both pathogens remains high. *Mucor rouxii* elicits dimorphism morphogenetic phenomenon: mycelium or yeast cells based on physiological conditions and influence of growth factors (Lucio et al. 2000). This interchanging mechanism play major role during host infection by *M. rouxii*. In recent years, the wide spread use of antibiotics have caused the emergence of antibiotic resistance *Aspergillus* sp. which has became a serious threat to immunosupressed patients (Van Der Linden et al. 2011). In the present study, a sesquiterpenes derivative with distinct antifungal activity patterns and relatively low toxicity was reported. High relatively lower toxicity of terpenes can be attributed to their biosynthetic pathway mediated structural changes (Souza et al. 2014; Wang et al. 2016). The results of MIC against these clinically significant pathogens have proved the capability of the metabolites for clinical use. Further toxicity works were performed on *C. elegans* to ensure these compounds *in vivo* efficacy. Egg hatching and young to adult conversion was significantly blocked in *C. elegans* which is an additional result which was observed after 40 hours of sesquiterpenes treatment. Using *C. elegans – C. albicans* model system, we investigated the *in vivo* antifungal activity of sesquiterpenes extracted from deep-sea fungus *Ascotricha* sp. To validate our *in vitro* analysis the sesquiterpenes promoted the fungus infected nematodes survival. Interestingly, few *C. albicans* reverted from pathogenic filamentous forms to vegetative yeast forms in presence of secondary metabolites. To ensure their survival under harsh external conditions, the pathogenic yeast might have devised a mechanism to adapt to drastic changes in environmental conditions. Thus it can switch between different phenotypic forms which contribute to its virulence. The switching from commensal to pathogenic phase has been widely thought to be associated with the phenotypic plasticity of *C. albicans*. It can grow in several morphological forms including unicellular yeast-form, elongated hyphae and pseudohyphae (Huang 2012). This suggests that secondary metabolites indeed function in improving nematode fitness via targeting pathogenic fungus (*C. albicans*). Secondly, the resistance towards pathogens were transferred to offsprings and healthy ones were generated. This functioning of metabolites in attenuating pathogenic fungus sheds new light on the antifungal drug discovery. The 5-HT_2C_ receptor is a novel drug target to treat several neural disorders. Recently, the crystal structures of the 5-HT_2C_ was solved. Many receptor subtypes have been screened and completely identified, consisting of three members of the 5-HT_2_ subclass characterized by close sequences homologies (Hoyer et al. 2002). It was elucidated that 5-HT_2C_ is a G protein-coupled receptor which constitutes only for CNS (Higgins et al. 2013). Previous studies on the interaction of 5-HT_2C_ with ergotamine (5-HT_2C_ agonist) also suggests a significant identity in terms of the binding pocket residues. This construes the fact the ascotricin-A binds to the similar crucial residues as that of ergotamine, thereby acts as a serotonin receptor agonist. The simulation analysis pinpoints amino acid difference at various positions in the 5-HT_2C_ receptor that impacts translation of molecules for neural related disorders.

## Conclusion

The present study is the first to report a piezotolerant *Ascotricha* sp. from deep-sea. The important characteristic of this fungus was its novel piezotolerance, production of broad-spectrum anti-fungal metabolite sesquiterpenes and a serotonin 5-HT_2C_ receptor agonist ascotricin. The *in-vivo* antifungal analysis confirmed that sesquiterpenes is a potent antifungal drug candidate. The *in silico* molecular docking and simulation studies confirmed ascotricin as a novel serotonin receptor agonist. These results confirm that deep-sea fungus *Ascotricha* sp. extract could be exploited as a potential source for antifungal compounds and 5-HT_2C_ receptor-specific agonist which can be used for several medical applications.
