# Supplementary figures and images for: Studies on the antifungal and serotonin receptor agonist activities of the secondary metabolites from piezotolerant deep-sea fungus Ascotricha sp
Source: Mycology. 2018 Nov 21;10(2):92–108. doi: 10.1080/21501203.2018.1541934 (PMC6493281; doi:10.1080/21501203.2018.1541934)

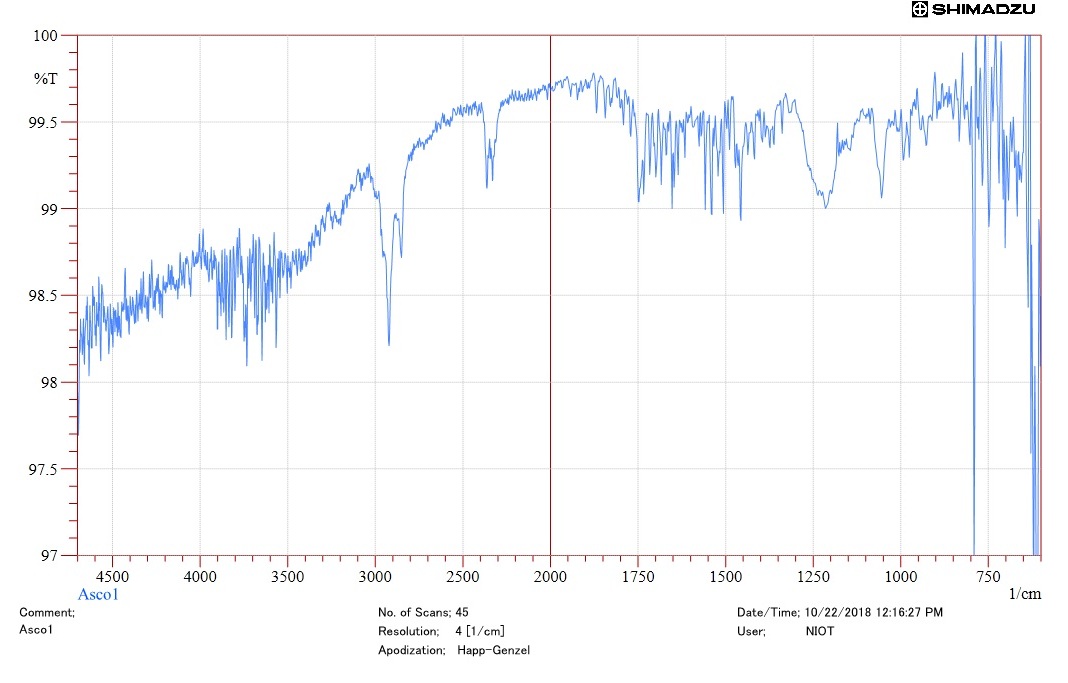

Supplement: Supplemental Material [file TMYC_A_1541934_SM8186.zip › Supplementary 1_ASCO1_FTIR.jpg]

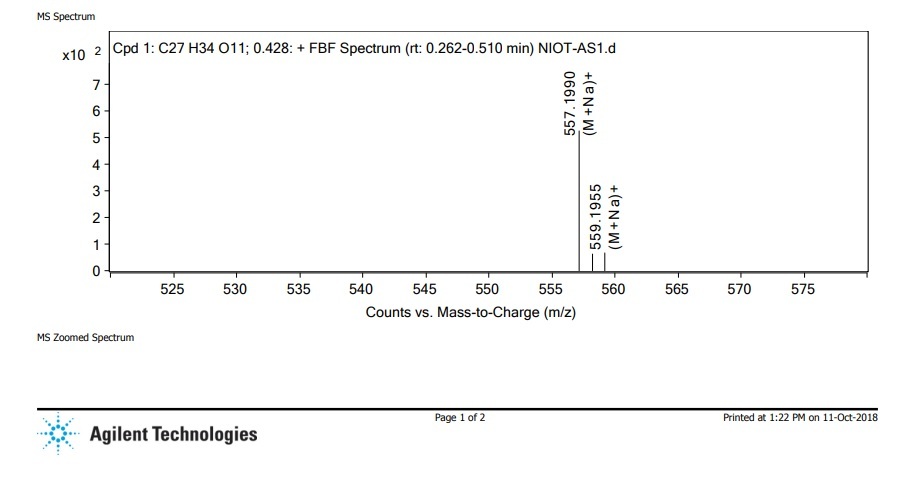

Supplement: Supplemental Material [file TMYC_A_1541934_SM8186.zip › Supplementary 2_ASCO_MS.jpg]
